# scHiCTools: A computational toolbox for analyzing single-cell Hi-C data

**DOI:** 10.1371/journal.pcbi.1008978

**Published:** 2021-05-18

**Authors:** Xinjun Li, Fan Feng, Hongxi Pu, Wai Yan Leung, Jie Liu

**Affiliations:** 1 Department of Statistics, University of Michigan, Ann Arbor, Michigan, United States of America; 2 Department of Computational Medicine and Bioinformatics, University of Michigan, Ann Arbor, Michigan, United States of America; 3 College of Literature Science, and the Arts, University of Michigan, Ann Arbor, Michigan, United States of America; Johns Hopkins University, UNITED STATES

## Abstract

Single-cell Hi-C (scHi-C) sequencing technologies allow us to investigate three-dimensional chromatin organization at the single-cell level. However, we still need computational tools to deal with the sparsity of the contact maps from single cells and embed single cells in a lower-dimensional Euclidean space. This embedding helps us understand relationships between the cells in different dimensions, such as cell-cycle dynamics and cell differentiation. We present an open-source computational toolbox, **scHiCTools**, for analyzing single-cell Hi-C data comprehensively and efficiently. The toolbox provides two methods for screening single cells, three common methods for smoothing scHi-C data, three efficient methods for calculating the pairwise similarity of cells, three methods for embedding single cells, three methods for clustering cells, and a build-in function to visualize the cells embedding in a two-dimensional or three-dimensional plot. **scHiCTools**, written in Python3, is compatible with different platforms, including Linux, macOS, and Windows.

## Author summary

Single-cell Hi-C contact maps describe the numbers of interactions among genomic loci across the entire genome, and provide researchers 3D chromatin organization in each cell. There are growing demands for an easy and fast way to analyze and visualize single-cell Hi-C data, and analyzing single-cell Hi-C data exposes several inherent data analysis challenges. To move beyond existing computational tools and methods to analyze and visualize single-cell Hi-C data, we present a software package, scHiCTools, which is implemented in Python. The software package provides researchers a collection of methods to investigate the cell-to-cell similarity based on their 3D chromatin organization, cluster cells into groups accordingly, and visualize cells in two-dimensional or three-dimensional scatter plots. In this paper, we provide an overview of scHiCTools’ structure and capabilities. We then apply scHiCTools to several single-cell Hi-C datasets to benchmark the performance of the methods provided in our toolbox, and present some plots generated using the software package.

This is a *PLOS Computational Biology* Software paper.

## Introduction

Recent single-cell Hi-C sequencing (scHi-C) technologies profile three-dimensional (3D) chromatin contact maps in individual cells, allowing us to characterize chromatin organization dynamics and cell-to-cell heterogeneity [1–3]. However, the interpretation of scHi-C data exposes several inherent data analysis challenges [4]. First, unlike RNA-seq data and ATAC-seq data which are vectors of *m*-dimensional measures, Hi-C data are essentially symmetric matrices of *m* × *m*-dimensional pairwise measures, where the number of genomic loci *m* is usually more than tens of thousands, depending on the resolution of the contact maps. Second, scHi-C analysis suffers from high-dimensionality, the sparsity of the contact maps, and sequencing noise. Typically in a scHi-C experiment, up to a few thousands of single cells are profiled, whereas the number of contacts in each cell ranges from a few thousands to hundreds of thousands. Third, single cells in one experiment usually reside in a low-dimensional manifold, such as a circular cell cycle structure or a bifurcation differentiation structure. Thus, proper embedding of scHi-C data in a low-dimensional Euclidean space is vital in scHi-C data analysis.

In a previous exploratory study [4], different similarity methods [5–9] have been applied to scHi-C data from *n* single cells, and coupled with multidimensional scaling (MDS) to project the *n* single cells into a low-dimensional Euclidean space. Among these methods, HiCRep [5] yields reasonable similarity measures and satisfactory embedding of the single cells, but its *O*(*n*^2^) computational complexity makes it impractical when the number of cells is large. In addition, this proof-of-concept study [4] did not provide any software implementation to embed scHi-C data, let along upstream analysis such as screening single cells and smoothing contact maps, and downstream analysis such as clustering and visualization.

In this work, we implemented a versatile **scHiCTools** which includes many common approaches in the entire workflow of analyzing single-cell Hi-C data. In particular, we implemented three similarity measures, including a faster version of HiCRep, a new “InnerProduct” approach, and another efficient Hi-C similarity measure named Selfish [10]. Among the three methods implemented, InnerProduct provides the most efficient and satisfactory similarity measure. Benchmarking experiments demonstrate that the new InnerProduct approach runs thousands of times faster than the original HiCRep, and produces comparably accurate projection. To deal with the sparsity in scHi-C data, different smoothing approaches are implemented, including linear convolution, random walk, and network enhancing [11]. Among the three approaches, linear convolution appears to be most effective for smoothing contact maps in our experiments. In addition to the computational components, our toolbox supports different input file formats, diagnostic summary plots, and flexible projection plots. Our open-source toolbox, **scHiCTools**, as the first toolbox of such kind, can be useful for analyzing scHi-C data.

## Design and implementation

### Overview

**Fig 1:**
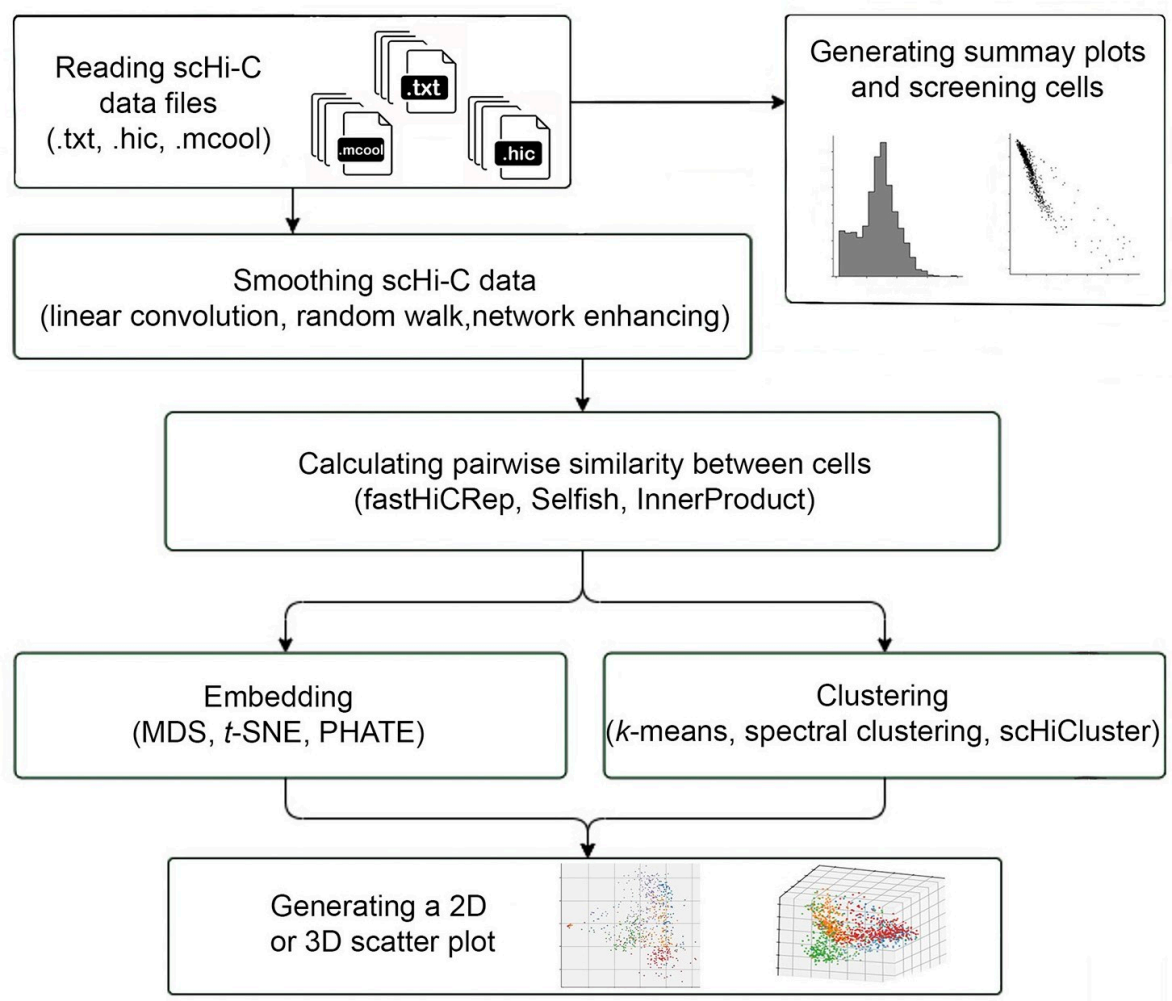
The workflow of scHiCTools. The workflow of scHiCTools includes five steps: (1) reading input single-cell data in .txt, .hic, or .cool format, generating the summary plots of the cells, and screening cells based on their contact number and contact distance profile, (2) smoothing the scHi-C contact maps using linear convolution, random walk, or network enhancing, (3) calculating the pairwise similarity between cells using fastHiCRep, InnerProduct, or Selfish, (4) embedding or clustering the cells in a low-dimensional Euclidean space using dimension reduction methods, and (5) visualizing the two-dimensional or three-dimensional embedding in a scatter plot.

Our **scHiCTools** implements commonly used approaches to analyze single-cell Hi-C data. The key component of the toolbox is a number of dimension reduction approaches which takes a number of single cells’ contact maps as input, and embeds the cells in a low-dimensional Euclidean space. The toolbox also provides a number of built-in auxiliary functions for flexible and interactive visualization. The entire workflow of scHiCTools, illustrated in Fig 1, includes five steps: (1) reading single-cell data in .txt, .hic, or .cool format, generating diagnostic summary plots, and screening cells by their contact number and contact distance profile, (2) smoothing scHi-C contact maps using linear convolution, random walk, or network enhancing, (3) calculating pairwise similarity between cells using fastHiCRep, InnerProduct, or Selfish, (4) embedding or clustering the cells in a low-dimensional space using dimension reduction methods, and (5) visualizing the two-dimensional or three-dimensional embedding in a scatter plot. Except for the two pairwise similarity calculation methods, fastHiCRep and InnerProduct, other methods are implemented as originally stated.

### Loading data and screening cells

**Fig 2:**
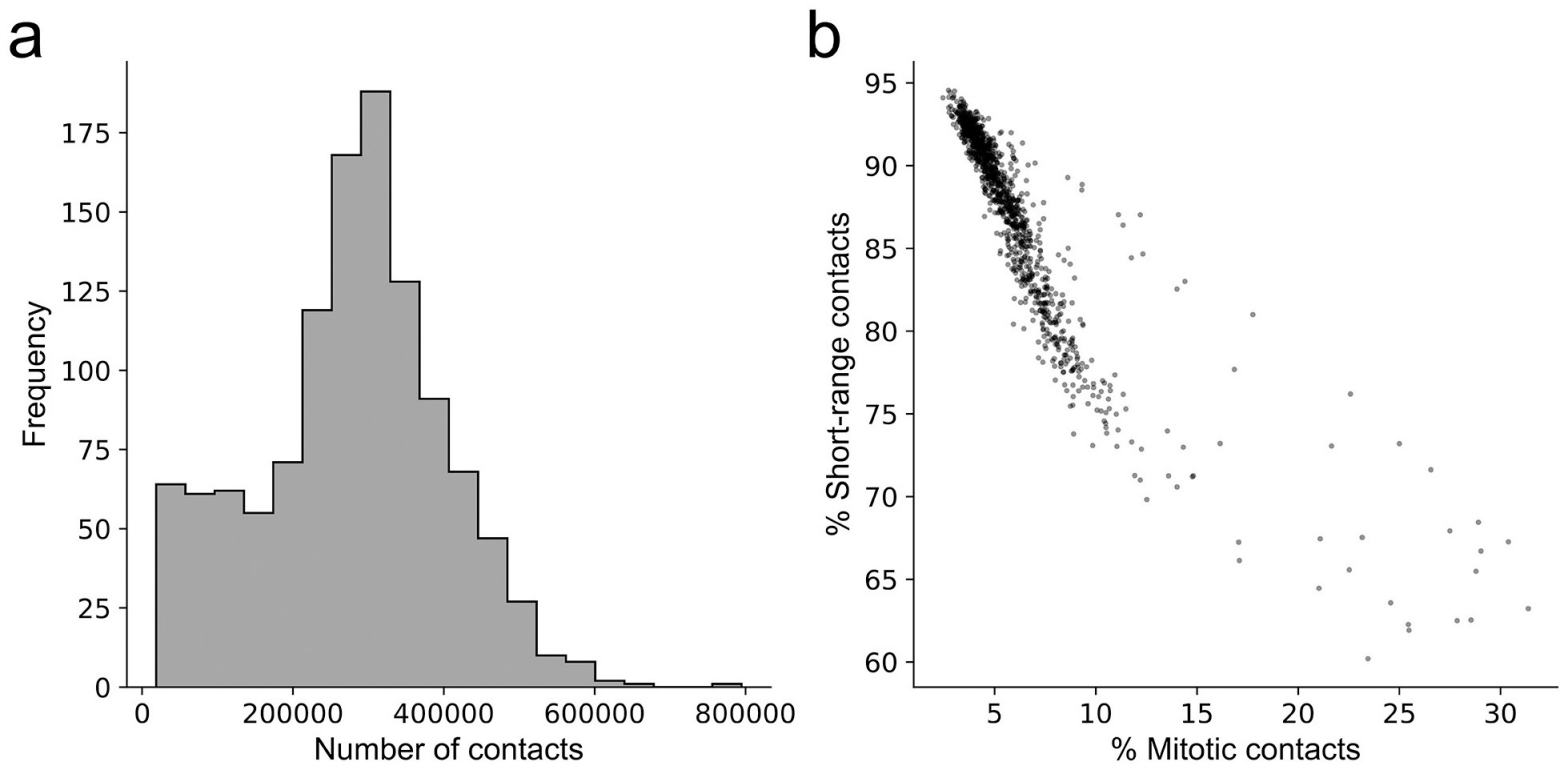
Summary plots for examining the quality of input scHi-C data. (a) A histogram of contact numbers in the individual cells. (b) A scatter plot showing the percentage of short-range contacts (<2 Mb) versus the percentage of contacts at the mitotic band (2 ∼ 12 Mb) in individual cells.

Users can load scHi-C data in different file formats, including .hic files, .cool files, and sparse contact matrices in text files. When users choose to load sparse matrices in text files, they are able to customize each column in the text files, and specify additional information including reference genome and the resolution of the contact maps. In addition, **scHiCTools** supports parallel file loading on a multi-core processor. After loading the data files, **scHiCTools** allows users to plot two summary plots to examine the quality of the loaded single-cell Hi-C data, namely a histogram of contact numbers, and a scatter plot of cells with the proportion of short-range contacts (<2 Mb) versus the proportion of the contacts at the mitotic band (2 ∼ 12 Mb) (Fig 2). A low-quality contact map is characterized by a low number of contacts and a relatively high proportion of short-range contacts. Users can further remove the low-quality cells by thresholding the number of contacts and the proportion of short-range contacts in the cells.

### Smoothing contact maps

Smoothing single cell contact maps is necessary when they are sparse, especially in a comparative analysis. Our toolbox **scHiCTools** implements three smoothing approaches.

**Linear convolution** is essentially a two-dimensional convolution filter with equal weights in every position, which can be viewed as smoothing over neighboring bins in Hi-C contact maps. For example, original HiCRep [5] uses a parameter *h* to describe a (2*h* + 1) × (2*h* + 1) convolution filter, i.e., *h* = 1 indicating a 3 × 3 kernel with each element $\frac{1}{9}$.

**Random walk** [12] is another approach to smooth chromatin contact maps. Unlike linear convolution which takes information from neighbors on the contact map, random walk captures the signals from a global setting as follows. Let *W* be the input *m* × *m* contact matrix. Random walk updates *W* by *W*^(*t*)^ = *W*^(*t*−1)^ ⋅ *B* in the *t*-th iteration, where *W*^(0)^ = *W*, and $B_{ij}=\frac{W_{ij}}{\sum_{j=1}^{m}W_{ij}}$. The matrix *B* is the input matrix divided by its row sum (i.e., every row of *B* sum up to 1). We can write *B* as *B* = *D*^−1^ ⋅ *W*, where $D=diag\{\sum_{j=1}^{m}W_{1j},\sum_{j=1}^{m}W_{2j},\cdots ,\sum_{j=1}^{m}W_{mj}\}$. After *t* steps of update, *W*^(*t*)^ = *W* ⋅ *B*^*t*^ is the output matrix after smoothing.

**Network enhancing** [11] is a special type of random walk which enhances network signals by increasing gaps between leading eigenvalues of a doubly stochastic matrix (DSM, the sum of each row and the sum of each column are both 1). To get a DSM, Knight-Ruiz (KR) normalization [13] is applied to the original contact matrix *W*, i.e., finding a vector *a*, such that *W*′ = *aWa*^⊤^ is a DSM. Network enhancing makes the partition of contact maps more prominent, and enhances boundaries of topologically associated domains (TADs) in chromatin contact maps [11].

### Calculating pairwise similarity

Hi-C contact maps are *m* × *m*-dimensional measures, where *m* is the number of genomic loci. By calculating pairwise similarity among the cells, we avoid dealing with the high-dimensional measure in the following embedding and clustering steps. Our toolbox **scHiCTools** includes the following three approaches for calculating pairwise similarity among the single cells.

**InnerProduct** calculates the pairwise similarity matrix among cells in two steps. The first step is “scaling”, which directly sets the first *s* *z*-normalized strata of one cell’s contact map as a feature vector for the cell. Because we only need to apply this step to the individual cells sequentially, this scaling step has an *O*(*n*) time complexity. Denote *v*_*i*_ = [*v*_*i*,1_, *v*_*i*,2_, ⋯, *v*_*i*,*m*−*i*_]^⊤^ to be the *i*-th stratum of the chromosome, *i* from 1 to *s*. *v*_*i*,*k*_ = *W*_*k*,*k*+*i*_ is the *k*-th element of *v*_*i*_, where *W* is the contact matrix of a chromosome. Subsequently, *z*-normalization is applied to each *v*_*i*_ to get a zero-mean and unit-variance vector $v_{i}^{\prime }$. By concatenating all strata, the feature vector for each contact map is $V=[v_{1}^{\prime },v_{2}^{\prime },...,v_{s}^{\prime }{]}^{⊤}$. The second step is “multiplication”, which calculates an inner product of the *n* feature vectors from the *n* cells to obtain the *n* × *n* similarity matrix. The second step has an *O*(*n*^2^) time complexity, but this step can be implemented efficiently with matrix multiplication in NumPy. Namely, we directly calculate the inner product of the two feature vectors *V*_*x*_ and *V*_*y*_ of map *x* and map *y* as their similarity,

$$\begin{array}{c} r_{xy}=\sum_{i=1}^{s}\frac{\left\langle \left(v_{i}^{x}-\bar{v_{i}^{x}}\right),\left(v_{i}^{y}-\bar{v_{i}^{y}}\right)\right\rangle }{\sqrt{var\left(v_{i}^{x}\right)var\left(v_{i}^{y}\right)}}=\left\langle [\begin{array}{c} \frac{v_{1}^{x}-\bar{v_{1}^{x}}}{\sqrt{var(v_{1}^{x})}}\\ \frac{v_{2}^{x}-\bar{v_{2}^{x}}}{\sqrt{var(v_{2}^{x})}}\\ \ldots \\ \frac{v_{s}^{x}-\bar{v_{s}^{x}}}{\sqrt{var(v_{s}^{x})}} \end{array}],[\begin{array}{c} \frac{v_{1}^{y}-\bar{v_{1}^{y}}}{\sqrt{var(v_{1}^{y})}}\\ \frac{v_{2}^{y}-\bar{v_{2}^{y}}}{\sqrt{var(v_{2}^{y})}}\\ \ldots \\ \frac{v_{s}^{y}-\bar{v_{s}^{y}}}{\sqrt{var(v_{s}^{y})}} \end{array}]\right\rangle . \end{array}$$ (1)

Run time of InnerProduct is also linear with respect to the length of *V*, which depends on the product of *m* and *s*, where *m* is the size of the contact map, and *s* is the number of strata from the diagonal considered in the calculation. Taking the average of *r*_*xy*_ over all chromosomes keeps the matrix positive definite, and gives us an overall kernel matrix of all the cells in the input. In practice, although taking the median may make the matrix no longer positive definite, it makes the similarity more stable and robust to noisy measurements.

**fastHiCRep** is a faster implementation of the original HiCRep approach [5]. Original HiCRep [5] calculates *s* stratum-adjusted correlation coefficients (SCCs) of the *s* strata near the diagonal of two contact maps, namely

$$\begin{array}{c} \text{SCC}=\frac{\sum_{i=1}^{s}r_{i}\left(m-i\right)\sqrt{var\left(v_{i}^{x}\right)var\left(v_{i}^{y}\right)}}{\sum_{i=1}^{s}\left(m-i\right)\sqrt{var\left(v_{i}^{x}\right)var\left(v_{i}^{y}\right)}}, \end{array}$$ (2)

where *v*_*i*_ is the *i*-th stratum of the chromosome, *m* − *i* is the length of *v*_*i*_, and *r*_*i*_ is the Pearson’s correlation coefficient of $v_{i}^{x}$ and $v_{i}^{y}$.

We have $var(v_{i})=\frac{\sum_{t=1}^{m-i}(v_{i,t}-\bar{v_{i}}{)}^{2}}{m-i}$, then

$$\begin{array}{c} r_{i}=\frac{\sum_{t=1}^{m-i}\left(v_{i,t}^{x}-\bar{v_{i}^{x}}\right)\left(v_{i,t}^{y}-\bar{v_{i}^{y}}\right)}{\sqrt{\sum_{t=1}^{m-i}{\left(v_{i,t}^{x}-\bar{v_{i}^{x}}\right)}^{2}\sum_{t=1}^{m-i}{\left(v_{i,t}^{y}-\bar{v_{i}^{y}}\right)}^{2}}}=\frac{\left\langle \left(v_{i}^{x}-\bar{v_{i}^{x}}\right),\left(v_{i}^{y}-\bar{v_{i}^{y}}\right)\right\rangle }{\left(m-i\right)\sqrt{var\left(v_{i}^{x}\right)var\left(v_{i}^{y}\right)}}. \end{array}$$ (3)

Note that the denominator term of *r*_*i*_ can be cancelled out with $(m-i)\sqrt{var(v_{i}^{x})var(v_{i}^{y})}$ in the numerator of SCC in Eq (2). We can write SCC between contact maps *x* and *y* as

$$\begin{array}{c} SCC_{xy}=\frac{\sum_{i=1}^{s}\left\langle \left(v_{i}^{x}-\bar{v_{i}^{x}}\right),\left(v_{i}^{y}-\bar{v_{i}^{y}}\right)\right\rangle }{\sum_{i=1}^{s}\left(m-i\right)\sqrt{var\left(v_{i}^{x}\right)var\left(v_{i}^{y}\right)}}=\frac{\left\langle vec^{x},vec^{y}\right\rangle }{\left\langle var^{x},var^{y}\right\rangle } \end{array}$$ (4)

where $vec^{x}={\left[\begin{array}{c} v_{1}^{x}-\bar{v_{1}^{x}},\cdots ,v_{s}^{x}-\bar{v_{s}^{x}} \end{array}\right]}^{⊤},$ $vec^{y}={\left[\begin{array}{c} v_{1}^{y}-\bar{v_{1}^{y}},\cdots ,v_{s}^{y}-\bar{v_{s}^{y}} \end{array}\right]}^{⊤}$ and $var^{x}={\left[\begin{array}{c} \sqrt{\left(m-1)var(v_{1}^{x}\right)},\cdots ,\sqrt{\left(m-s)var(v_{s}^{x}\right)} \end{array}\right]}^{⊤},$ $var^{y}={\left[\begin{array}{c} \sqrt{\left(m-1)var(v_{1}^{y}\right)},\cdots ,\sqrt{\left(m-s)var(v_{s}^{y}\right)} \end{array}\right]}^{⊤}$.

From above, SCC can be calculated efficiently because for each cell, cell *x* for example, *vec*^*x*^ and *var*^*x*^ only need to be calculated once. The vectors required in both the numerator and the denominator are generated sequentially for individual cells with an *O*(*n*) time complexity. Note that both the numerator and the denominator are inner products. Calculating the inner products has an *O*(*n*^2^) time complexity, but this step can be implemented efficiently with matrix multiplication in NumPy. However, there is one subtle difference between original HiCRep and our implemented fastHiCRep. In original HiCRep, if one chromatin contact in both cells *x* and *y* is zero, then this contact position will not be used in the calculation of SCC. Because HiCRep need to remove zeros and this operation is specific to the two cells to be compared, we need to calculate *r*_*i*_, $var(v_{i}^{x})$, and $var(v_{i}^{y})$ in Eq (2) $\left(\begin{array}{c} n\\ 2 \end{array}\right)$ times. Empirically, the SCC calculated by fastHiCRep and HiCRep did not differ too much. We randomly select a cell from Nagano dataset (cell 205 of Early-S stage) and compare the SCC scores using fastHiCRep versus HiCRep of other 1710 cells from Nagano dataset. The correlation coefficient of the SCC scores calculated by fastHiCRep and SCC scores calculated by HiCRep is 0.991.

The third similarity measure **Selfish** [10] was recently proposed for bulk Hi-C comparative analysis. It first uses a sliding window to obtain a number of rectangular regions along the diagonal of the contact map, and then counts overall contact numbers in each region. Then it generates a one-hot “fingerprint matrix” for each contact map. Finally, Gaussian kernels over the fingerprint matrices are calculated as similarities among the cells.

### Embedding and clustering

Although single cell measures are high-dimensional, the single cells usually reside in a low-dimensional manifold such as a circular cell cycle structure and a bifurcation differentiation structure. By embedding the cells in a lower-dimensional Euclidean space, we can easily explore the heterogeneity and structures among the single cells. **scHiCTools** includes three different dimension reduction methods that use pairwise similarity matrices among the cells to embed them in a low-dimensional Euclidean space. The three dimension reduction methods are as follows.

**MDS** (Multidimensional scaling) takes in a pairwise distance matrix evaluated in the original space, and embeds the data points in a lower-dimensional space which preserves the pairwise distance matrix. In our package, we use the classical MDS which finds the *p* dimensional embedding $X=[x_{1},x_{2},\cdots ,x_{n}{]}^{⊤}\in {\mathbb{R}}^{n\times p}$ that minimizes the loss function: *loss* = ‖*XX*^⊤^ − *G*‖_*F*_, where $G=-\frac{1}{2}(I_{n}-\frac{1}{n}11^{⊤})D(I_{n}-\frac{1}{n}11^{⊤})$, *D*_*ij*_ is the distance between *i*-th and *j*-th cells, and **1** denotes a column vector of all ones.

***t*-SNE** [14] embeds high-dimensional data in a low-dimensional space with an emphasis on preserving local neighborhood. *t*-SNE assumes that data points *x*_1_, ⋯, *x*_*n*_ in the high-dimensional space follow a Gaussian distribution, and the embedded points *y*_1_, ⋯, *y*_*n*_ follow a Student’s *t*-distribution. In the higher-dimensional space, the conditional probability of *x*_*i*_ picking *x*_*j*_ as its neighbor is $p_{j|i}=\frac{\text{exp}(-\|x_{i}-x_{j}{\|}^{2}/2\sigma_{i}^{2})}{\sum_{k\neq i}\text{exp}(-\|x_{i}-x_{k}{\|}^{2}/2\sigma_{i}^{2})}$. In our implementation, instead of computing the norm between two points, we directly use distances between two cells to calculate the similarity. The similarity between *x*_*i*_ and *x*_*j*_ is defined as the probability of picking *x*_*i*_ and *x*_*j*_ as neighbors, which is $p_{ij}=\frac{p_{j|i}+p_{i|j}}{2n}$. In the embedding space, since *y*_1_, ⋯, *y*_*n*_ ∼ *t*_1_, the probability of picking *y*_*i*_ and *y*_*j*_ as neighbors is $q_{ij}=\frac{(1+\|y_{i}-y_{j}{\|}^{2}{)}^{-1}}{\sum_{k\neq i}(1+\|y_{i}-y_{k}{\|}^{2}{)}^{-1}}$. The Kullback–Leibler divergence of the distribution of data points from the distribution of embedded points is $KL=\sum_{i\neq j}p_{ij}\text{log}\frac{p_{ij}}{q_{ij}}$. The embedding of the cells are optimized by minimizing the KL-divergence above.

**PHATE** (Potential of Heat-diffusion for Affinity-based Trajectory Embedding) [15] is a dimension reduction approach which preserves both local and global similarity. PHATE first calculates a local affinity matrix based on *k*-nearest neighbor distance *K*_*k*_, which captures the local structure of the data points. PHATE normalizes *K*_*k*_ using a Gaussian kernel to obtain a new matrix *P*, and then diffuses *P* by *t* steps to get a new matrix *P*^*t*^ which preserves the global structure of data points. Based on matrix *P*^*t*^, a potential representation of data can be calculated as *U*_*t*_ = −*log*(*P*^*t*^). Finally, PHATE applies non-metric MDS to *U*_*t*_ and generates low-dimensional embedding of the cells.

Clustering methods are desirable in the situation that the single cells come from discrete clusters rather than a continuous manifold. The following optional clustering methods are implemented in our toolbox.

***k*-means** assigns an observation to the cluster with the nearest cluster centroid, which is the mean of all observations belonging to the cluster. We use *k*-means++ [16] to initialize the centroids of clusters. Iterations of *k*-means algorithm renew the clusters by finding the points closest to the centroid in the last iteration, and update the centroid of each cluster by taking the mean of points in each cluster. Since *k*-means needs coordinates of cells in a Euclidean space to find the centroid of each cluster, we use MDS to embed the cells into a *l*-dimensional space first, and then perform *k*-means accordingly.

**Spectral clustering** [17] takes in a distance matrix of data points and divides *n* observations into *k* clusters. Spectral clustering directly takes in the distance matrix and constructs a similarity graph with Gaussian similarity function. Based on the similarity graph, spectral clustering calculates the Laplacian of the similarity graph, and projects the points into a *k*-dimensional space based on the first *k* eigenvectors of the graph Laplacian matrix. Finally, it uses *k*-means to divide the data points into *k* group.

**scHiCluster** [18] is a clustering method designed explicitly for single-cell Hi-C data. It uses convolution and random walk for smoothing and imputation, then converts the contact matrices to binary matrices. scHiCluster conducts principal component analysis for embedding, and then *k*-means for clustering.

### Visualization of embedding

**Fig 3:**
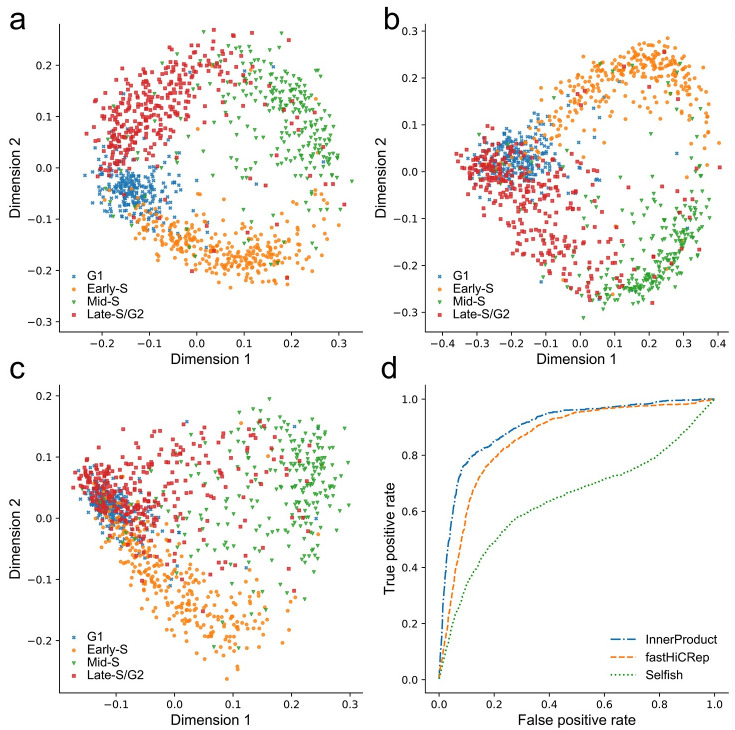
Two-dimensional scatter plots of the embedding from the three methods that calculate the similarity between contact matrices, including InnerProduct, fastHiCRep and Selfish. (Dataset: Nagano et al., 2017). (a) Two-dimensional projection from InnerProduct and MDS shows a clear circular pattern along the four stages of cell cycle. (b) Two-dimensional projection using fastHiCRep and MDS does not show clear separation between the four stages of cell cycle. (c) Two-dimensional projection using Selfish and MDS does not show clear separation between the four stages of cell cycle. (d) Evaluating the three embedding methods in a cell-cycle phasing task by ACROC. The ACROC values from InnerProduct, fastHiCRep and Selfish are 0.904, 0.858 and 0.642, respectively.

**scHiCTools** supports two-dimensional and three-dimensional plotting of the cell embedding. Fig 3 shows the scatter plots of the two-dimensional embedding of the cells in a cell cycle study [2]. In the situation that the cells reside on a three-dimensional manifold, a three-dimensional scatter plot of the cells can be visualized (S2 File). As a convenient option, an interactive scatter plot can be visualized, in which the cell label is displayed when the user’s mouse hovers (see S3 File). In order to use this feature, *ploty* is required on user’s device.

## Results

In this section, we apply the toolbox on a number of scHi-C datasets [2, 19], and benchmark the performance of different methods implemented. In addition to plotting the two-dimensional embedding of cells and examining whether the embedding is sensible, we benchmark the projection performance on a scHi-C dataset [2] with the average area under the curve of a circular ROC calculation (ACROC) proposed in a recent work [4]. ACROC measure ranges between 0 to 1. An embedding representing a better circular pattern produces a larger ACROC value. We record the run time of these methods to compare their efficiency. We evaluate the clustering performance with two criteria: normalized mutual information (NMI) [20] and adjusted rand index (ARI) [21] on another scHi-C dataset [19]. We have the following observations.

### InnerProduct is effective for calculating the pairwise similarity among single-cell Hi-C contact maps

When InnerProduct is coupled with MDS, it produces an accurate projection of single cells along four stages of cell cycle (Fig 3a), achieving an average area under a circular ROC calculation curve (ACROC) of 0.904, which is comparable to original HiCRep reported in the recent work [4]. Two-dimensional scatter plots from fastHiCRep and Selfish show a circular pattern along the four stages of cell cycle, but the separation between the stages is not as clear as that from InnerProduct (Fig 3b and 3c). The ACROC measure from fastHiCRep is 0.858 and the ACROC measure from Selfish is 0.642, which are both lower than that from InnerProduct (Fig 3d).

### PHATE and *t*-SNE produce satisfactory projections

**Fig 4:**
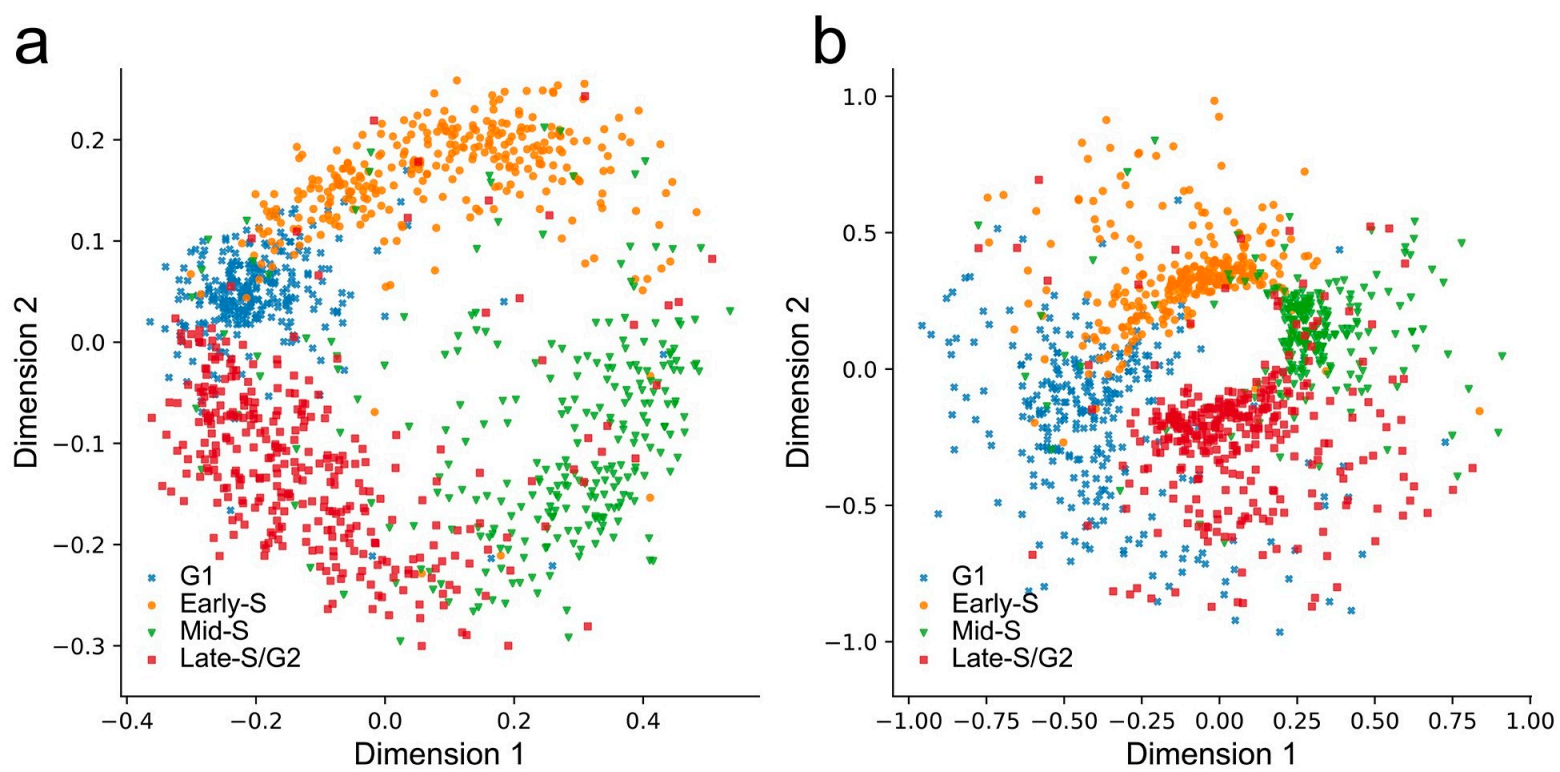
Two-dimensional embedding using different dimension reduction methods. (Dataset: Nagano et al., 2017). (a) Two-dimensional projection from InnerProduct/PHATE shows a circular pattern similiar to MDS projection (ACROC: 0.920). (b) Two-dimensional projection from InnerProduct/*t*-SNE shows a circular pattern (ACROC: 0.901).

Since MDS recovers global pairwise distance in its projection, it is unclear whether methods that preserve local pairwise distance (i.e., PHATE and *t*-SNE) can produce a better projection. Fig 4a and 4b show that PHATE and *t*-SNE are suitable embedding methods that project the smoothed contact maps into a lower-dimensional space while preserving a cell-cycle pattern. The ACROC measure of PHATE is 0.920, which is better than *t*-SNE (0.901) and MDS (0.904). Therefore, PHATE and *t*-SNE can be used alternatively, especially when faraway neighbors’ similarity cannot be properly evaluated.

### All three methods to calculate pairwise similarity are computationally efficient

The run time of InnerProduct, fastHiCRep, Selfish, and original HiCRep was measured on an Intel Xeon W-2175 CPU with a frequency of 2.50GHz (Table 1). Each experiment was replicated ten times, and the average run time was reported (Run time from different replicate experiments showed little variance. See S5 File for raw data). For embedding 1,000 cells randomly selected from data [2], three methods in our package, including InnerProduct, fastHiCRep, and Selfish, finished within minutes. In contrast, original HiCRep, also implemented in Python, took around five hours. For InnerProduct, we further measured the time spent in the first scaling step and in the second multiplication step. It was observed that the run time from the “scaling” step was linear in terms of cell number *n*, whereas the run time from the “multiplication” step was quadratic in terms of cell number *n*.

**Table 1:** Average run time (in seconds) of different methods as the number of cells vary. (Run time is averaged from 10 replicate experiments, performed on an Intel Xeon W-2175 CPU with a frequency of 2.50GHz).

# cells	HiCRep	fastHiCRep	InnerProduct			Selfish
			Scaling	Multiplication	Total	
100	267.91	0.05	0.05	0.03	0.08	0.11
200	1105.85	0.11	0.10	0.07	0.16	0.21
300	2470.76	0.19	0.14	0.10	0.24	0.36
400	4451.97	0.29	0.19	0.15	0.34	0.55
500	7025.12	0.41	0.25	0.23	0.48	0.80
600	10384.73	0.54	0.30	0.31	0.61	1.08
700	14366.02	0.70	0.37	0.44	0.80	1.41
800	18944.04	0.87	0.40	0.58	0.94	1.79
900	23878.24	1.10	0.49	0.82	1.30	2.21
1000	28335.26	1.32	0.55	0.98	1.53	2.70

### Linear convolution smoothing and random walk improve projection at high dropout rates

**Fig 5:**
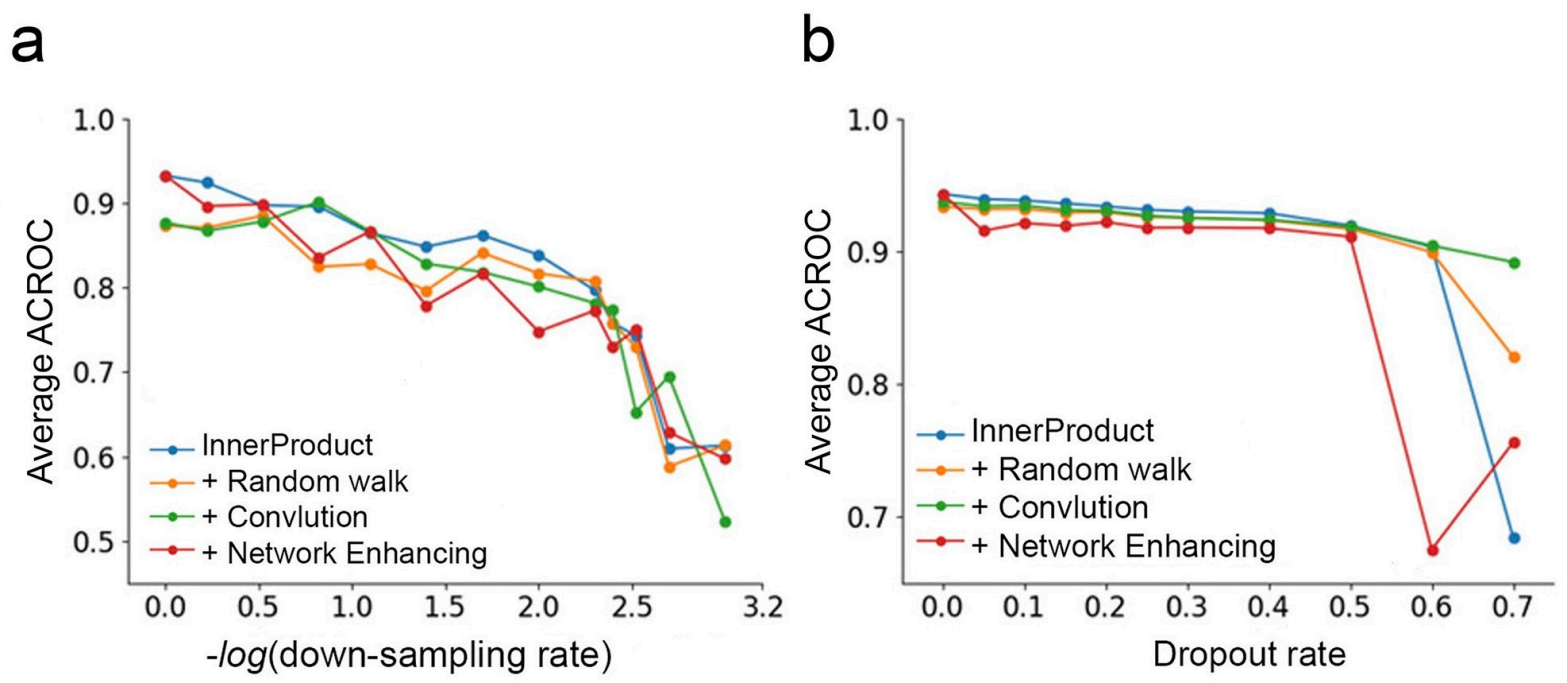
Average ACROC measures from InnerProduct without any smoothing, InnerProduct with random walk smoothing, InnerProduct with linear convolution, and InnerProduct with network enhancing. (a) When the dataset was sparsified with the first sparsification method, ACROC measures from the four approaches decreased when down-sampling rate increased. (b) When the dataset was sparsified with the second sparsification method, ACROC measures from the four approaches decreased when down-sampling rate increased, but at a high dropout rate (0.7), linear convolution’s ACROC remained high, whereas other methods’ ACROC dropped to below 0.9.

To examine the three smoothing approaches implemented in our toolbox, we sparsified the scHi-C dataset [2] with two methods. The first sparsification method is to remove 40% ∼ 99.9% of the contacts randomly from all genomic positions (i.e., contact number ranging from ∼200,000 to ∼500 in each cell). The second sparsification method is to simulate dropout events in sequencing data which discard contacts from 5% ∼ 60% of the genomic loci. With the sparsified datasets, we examined the quality of single cell embedding when different smoothing approaches were used. It was observed that none of the three smoothing methods, including linear convolution, random walk, and network enhancing, improved the embedding performance when the single cell contact maps were down-sampled by the first sparsification method (Fig 5a). The ACROC decreased from around 0.9 to around 0.6 as down-sampling rate changed from 1 to 0.05. Under the second sparsification method (dropout), linear convolution and random walk produced better single cell embedding, compared with the situation when no smoothing was used (Fig 5b). At a high dropout rate (0.7), linear convolution kept the average ACROC above 0.9, whereas other methods’ average ACROC’s dropped to below 0.9. Therefore, we recommend users use linear convolution to smooth scHi-C contact maps if they suspect dropout events exist moderately in their scHi-C data.

### scHiCluster produces better clustering results than InnerProduct coupled with spectral clustering or with *k*-means, but less efficient

Table 2 shows the performance of different clustering methods implemented in **scHiCTools** on the dataset of Collombet et al., 2020 [19]. We use all 750 mouse embryo cells at five differentiation stages in the study of Collombet et al., including the 1-cell, 2-cell, 4-cell, 8-cell, and 64-cell stages. We separate the cells into five clusters and evaluate the clusters using two evaluation measures, including normalized mutual information (NMI) [20], and adjusted rand index (ARI) [21]. A better clustering produces larger values of NMI and ARI. For random clustering, the values of NMI and ARI are close to 0. When the cluster agrees with the true label, NMI and ARI reach the upper bound 1. scHiCluster shows better NMI and ARI values than InnerProduct with *k*-means and InnerProduct with spectral clustering. InnerProduct with *k*-means and InnerProduct with spectral clustering are more efficient than scHiCluster. The run time of scHiCluster, InnerProduct with *k*-means, Selfish, and InnerProduct with spectral clustering was measured on an Intel Xeon W-2175 CPU with a frequency of 2.50GHz. Each experiment was replicated ten times, and the average run time was reported (Run time from different replicate experiments showed little variance. See S5 File for more details).

**Table 2:** The normalized mutual information (NMI), adjusted rand index (ARI), and run time of three clustering approaches. (Data: 750 embryo cells at five differentiation stages, including 1-cell, 2-cell, 4-cell, 8-cell and 64-cell stages, Collombet et al., 2020).

	NMI	ARI	Average run time (in seconds)
scHiCluster	0.266	0.259	114.421
InnerProduct+MDS+*k*-means	0.222	0.208	1.771
InnerProduct+spectral clustering	0.241	0.197	1.531

## Availability and future directions

Our scHiCTools is implemented in Python. The source code is available and maintained at Github: https://github.com/liu-bioinfo-lab/scHiCTools. This package is also available on PyPI python package manager. The current code runs under Python 3.7 or newer versions. Other dependency includes numpy, scipy, matplotlib, pandas, simplejson, six, and h5py. For the interactive scatter plot function, you need to have plotly installed. In the future, we will keep updating the toolbox with new scHi-C analysis algorithms, including new embedding methods such as UMAP and new clustering methods such as hierarchical clustering.

## Supporting information

S1 File Plots from different similarity measures and embedding methods applied to Nagano single-cell dataset. This zip file includes the plots of other combination of similarity measures (InnerProduct, fastHiCRep and Selfish) and embedding methods (MDS, *t*-SNE and PHATE).
(ZIP)

S2 File Three-dimensional scatter plots. This zip file includes the 3D scatter plots of different embedding methods (MDS, *t*-SNE and PHATE) applied to Nagano single-cell dataset.
(ZIP)

S3 File Interactive plots. This file includes examples of interactive 2D and 3D scatter plots of cells from Nagano et al.
(ZIP)

S4 File scHiCTools source code, documentation and test dataset. This zip file is a clone of scHiCTools public Git repository. To install from this file rather than from PyPI, please follow installation instructions in the readme file.
(ZIP)

S5 File Run time details. This PDF file includes the run time of similarity calculation methods and clustering methods.
(PDF)

S6 File Details of applying our toolbox to Flyamer et al. [1], Collombet et al. [19] and Ramani et al. [3] datasets. This PDF file includes the number of cells and the contacts numbers of Flyamer et al., Collombet et al. and Ramani et al. datasets, and their scatter plots.
(PDF)
