# Supplementary figures and images for: scHiCTools: A computational toolbox for analyzing single-cell Hi-C data
Source: PLoS Comput Biol. 2021 May 18;17(5):e1008978. doi: 10.1371/journal.pcbi.1008978 (PMC8162587; doi:10.1371/journal.pcbi.1008978)

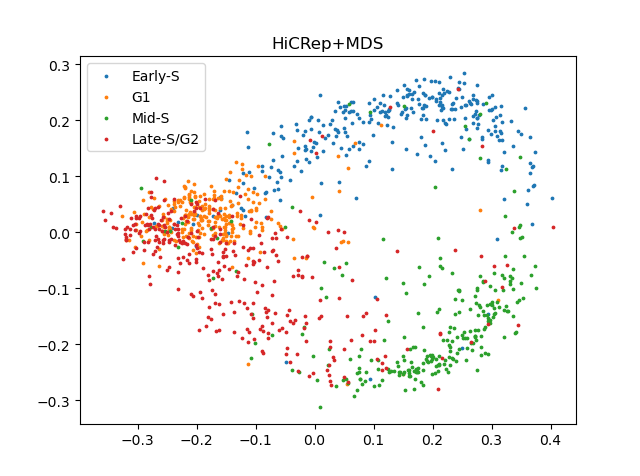

Supplement: S1 File — This zip file includes the plots of other combination of similarity measures (InnerProduct, fastHiCRep and Selfish) and embedding methods (MDS, t-SNE and PHATE). (ZIP) [file pcbi.1008978.s001.zip › S1/HiCRep+MDS.png]

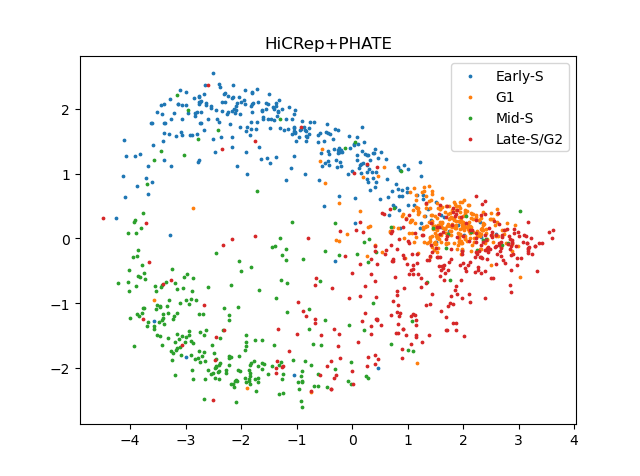

Supplement: S1 File — This zip file includes the plots of other combination of similarity measures (InnerProduct, fastHiCRep and Selfish) and embedding methods (MDS, t-SNE and PHATE). (ZIP) [file pcbi.1008978.s001.zip › S1/HiCRep+PHATE.png]

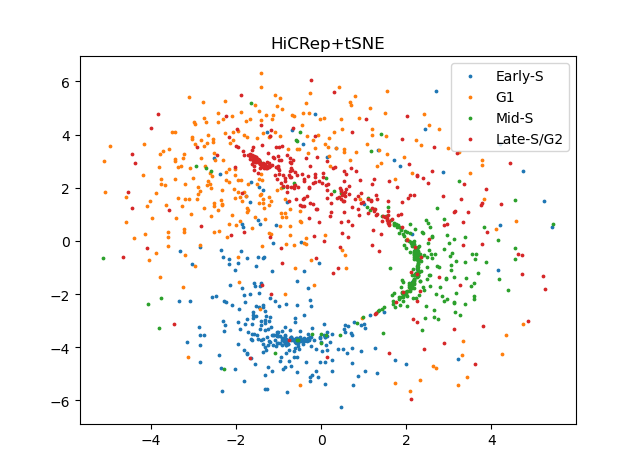

Supplement: S1 File — This zip file includes the plots of other combination of similarity measures (InnerProduct, fastHiCRep and Selfish) and embedding methods (MDS, t-SNE and PHATE). (ZIP) [file pcbi.1008978.s001.zip › S1/HiCRep+tSNE.png]

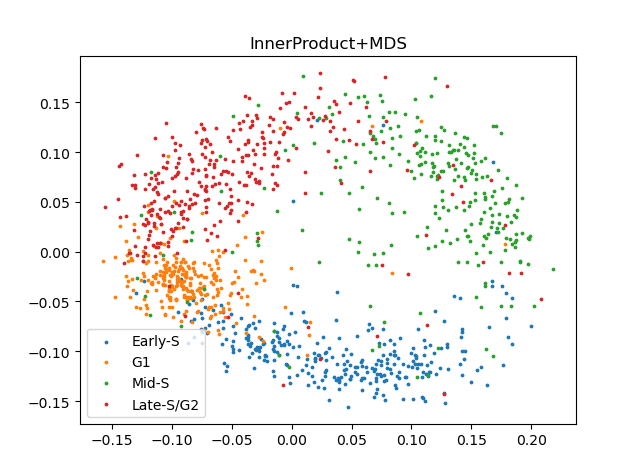

Supplement: S1 File — This zip file includes the plots of other combination of similarity measures (InnerProduct, fastHiCRep and Selfish) and embedding methods (MDS, t-SNE and PHATE). (ZIP) [file pcbi.1008978.s001.zip › S1/InnerProduct+MDS.png]

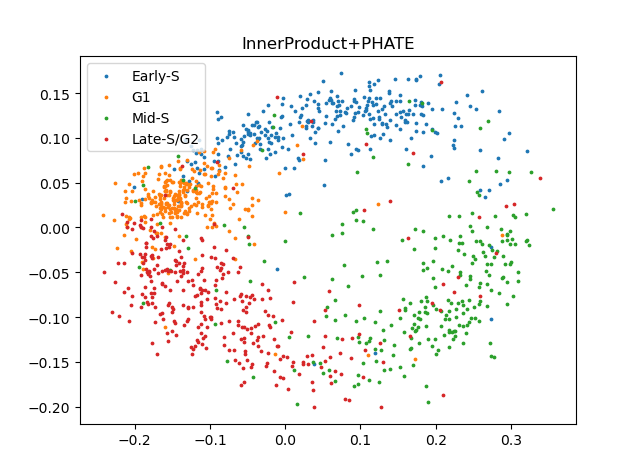

Supplement: S1 File — This zip file includes the plots of other combination of similarity measures (InnerProduct, fastHiCRep and Selfish) and embedding methods (MDS, t-SNE and PHATE). (ZIP) [file pcbi.1008978.s001.zip › S1/InnerProduct+PHATE.png]

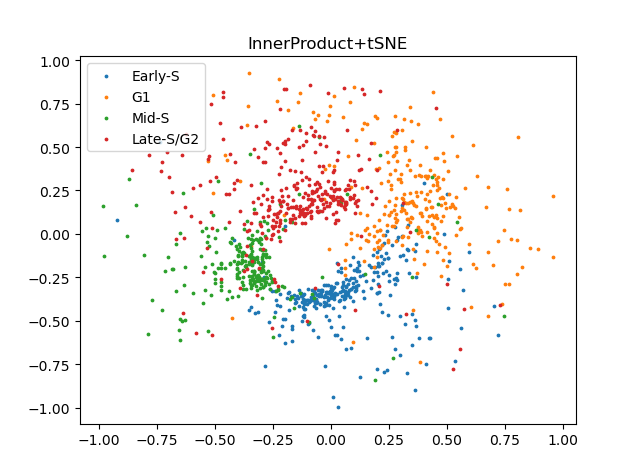

Supplement: S1 File — This zip file includes the plots of other combination of similarity measures (InnerProduct, fastHiCRep and Selfish) and embedding methods (MDS, t-SNE and PHATE). (ZIP) [file pcbi.1008978.s001.zip › S1/InnerProduct+tSNE.png]

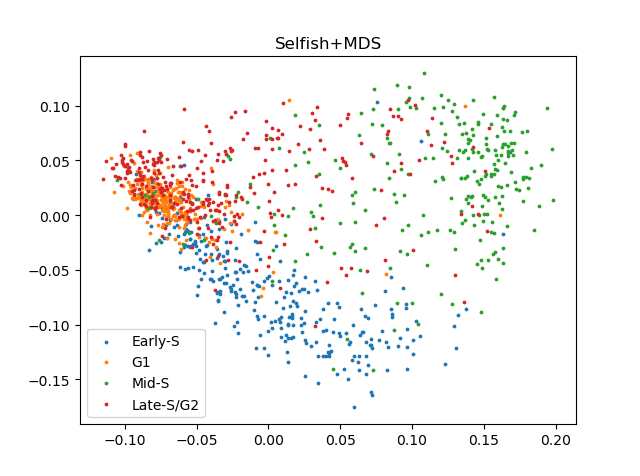

Supplement: S1 File — This zip file includes the plots of other combination of similarity measures (InnerProduct, fastHiCRep and Selfish) and embedding methods (MDS, t-SNE and PHATE). (ZIP) [file pcbi.1008978.s001.zip › S1/Selfish+MDS.png]

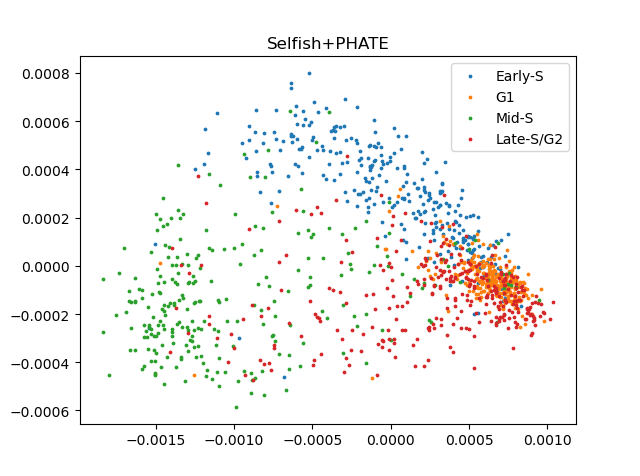

Supplement: S1 File — This zip file includes the plots of other combination of similarity measures (InnerProduct, fastHiCRep and Selfish) and embedding methods (MDS, t-SNE and PHATE). (ZIP) [file pcbi.1008978.s001.zip › S1/Selfish+PHATE.png]

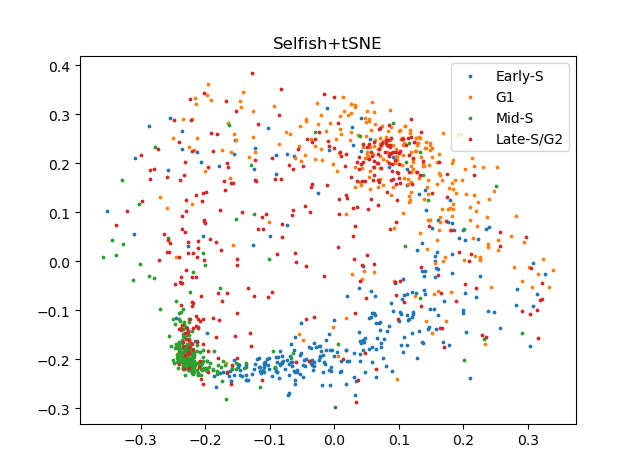

Supplement: S1 File — This zip file includes the plots of other combination of similarity measures (InnerProduct, fastHiCRep and Selfish) and embedding methods (MDS, t-SNE and PHATE). (ZIP) [file pcbi.1008978.s001.zip › S1/Selfish+tSNE.png]

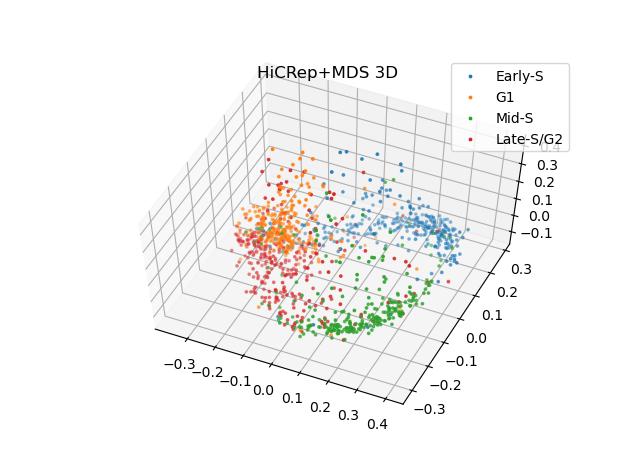

Supplement: S2 File — This zip file includes the 3D scatter plots of different embedding methods (MDS, t-SNE and PHATE) applied to Nagano single-cell dataset. (ZIP) [file pcbi.1008978.s002.zip › S2/HiCRep+MDS 3D.png]

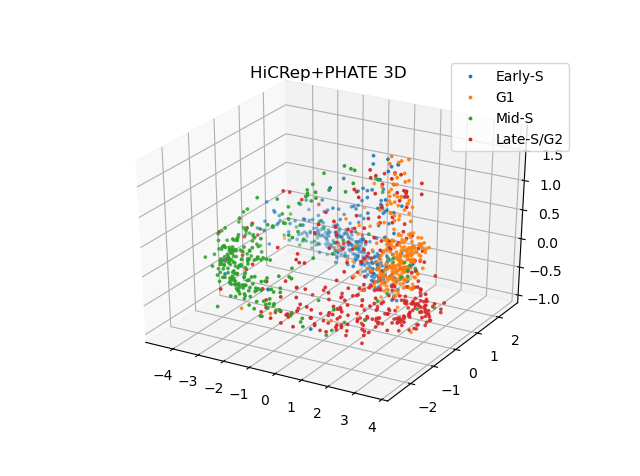

Supplement: S2 File — This zip file includes the 3D scatter plots of different embedding methods (MDS, t-SNE and PHATE) applied to Nagano single-cell dataset. (ZIP) [file pcbi.1008978.s002.zip › S2/HiCRep+PHATE 3D.png]

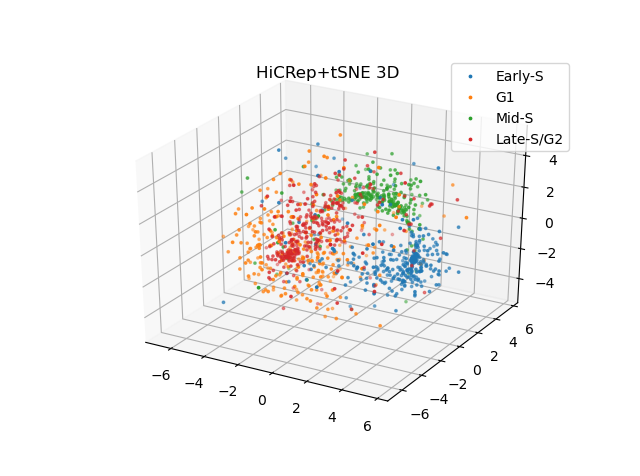

Supplement: S2 File — This zip file includes the 3D scatter plots of different embedding methods (MDS, t-SNE and PHATE) applied to Nagano single-cell dataset. (ZIP) [file pcbi.1008978.s002.zip › S2/HiCRep+tSNE 3D.png]

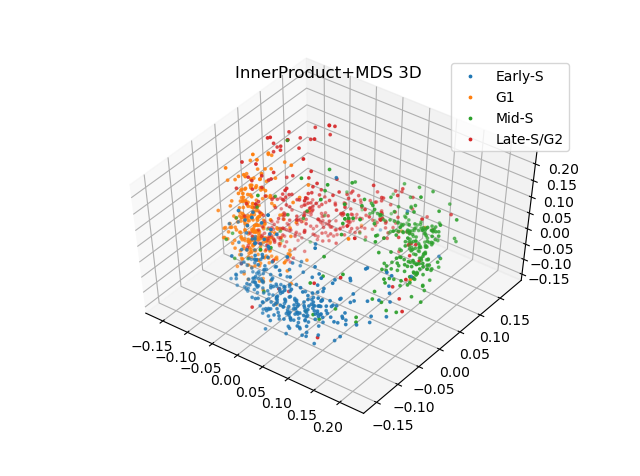

Supplement: S2 File — This zip file includes the 3D scatter plots of different embedding methods (MDS, t-SNE and PHATE) applied to Nagano single-cell dataset. (ZIP) [file pcbi.1008978.s002.zip › S2/InnerProduct+MDS_3D.png]

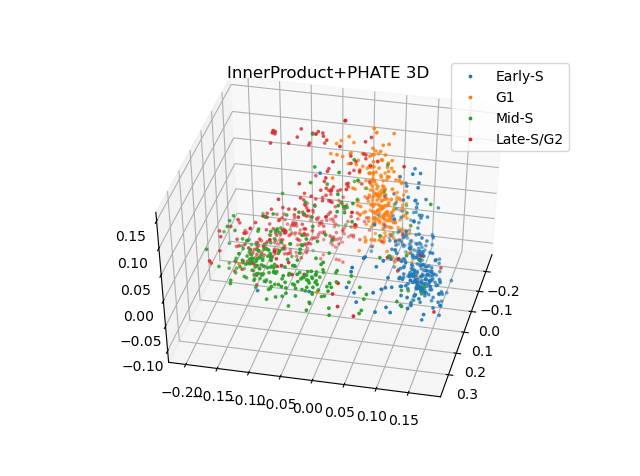

Supplement: S2 File — This zip file includes the 3D scatter plots of different embedding methods (MDS, t-SNE and PHATE) applied to Nagano single-cell dataset. (ZIP) [file pcbi.1008978.s002.zip › S2/InnerProduct+PHATE 3D.png]

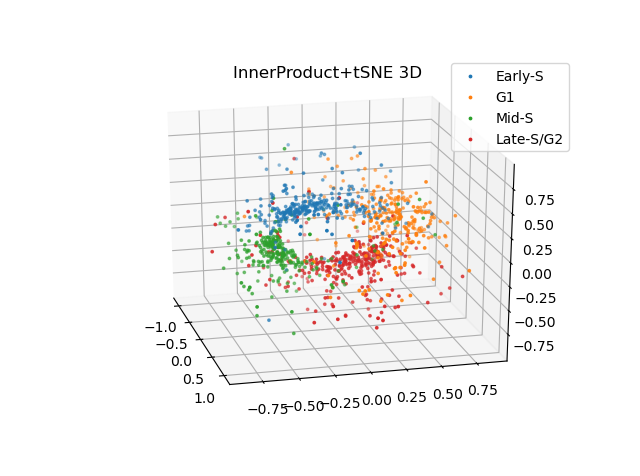

Supplement: S2 File — This zip file includes the 3D scatter plots of different embedding methods (MDS, t-SNE and PHATE) applied to Nagano single-cell dataset. (ZIP) [file pcbi.1008978.s002.zip › S2/InnerProduct+tSNE 3D.png]

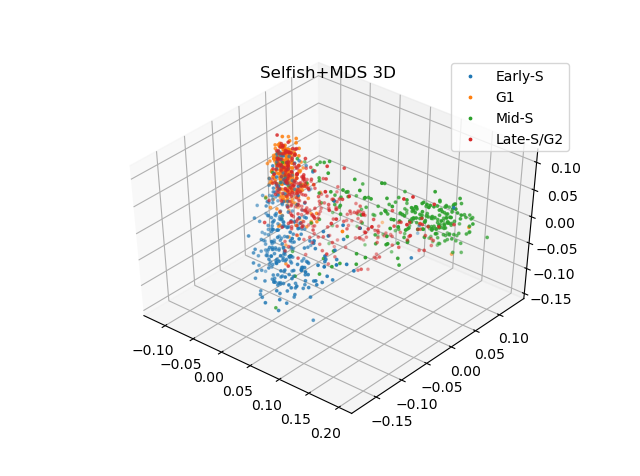

Supplement: S2 File — This zip file includes the 3D scatter plots of different embedding methods (MDS, t-SNE and PHATE) applied to Nagano single-cell dataset. (ZIP) [file pcbi.1008978.s002.zip › S2/Selfish+MDS 3D.png]

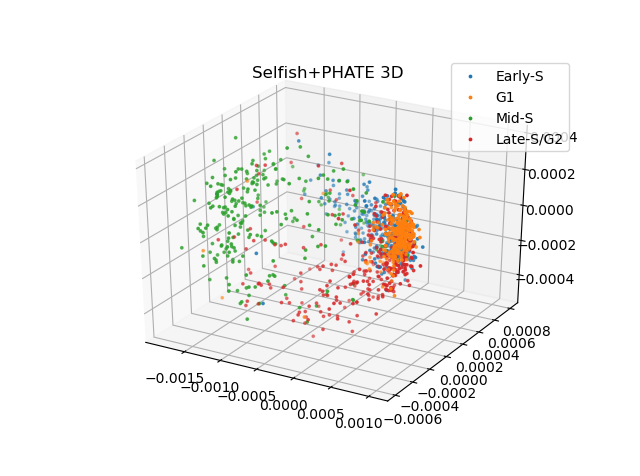

Supplement: S2 File — This zip file includes the 3D scatter plots of different embedding methods (MDS, t-SNE and PHATE) applied to Nagano single-cell dataset. (ZIP) [file pcbi.1008978.s002.zip › S2/Selfish+PHATE 3D.png]

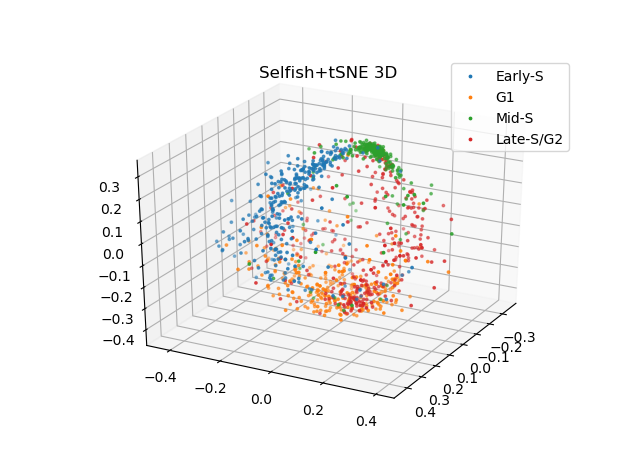

Supplement: S2 File — This zip file includes the 3D scatter plots of different embedding methods (MDS, t-SNE and PHATE) applied to Nagano single-cell dataset. (ZIP) [file pcbi.1008978.s002.zip › S2/Selfish+tSNE 3D.png]
