# Supplementary material for: scHiCTools: A computational toolbox for analyzing single-cell Hi-C data
Source: PLoS Comput Biol. 2021 May 18;17(5):e1008978. doi: 10.1371/journal.pcbi.1008978 (PMC8162587; doi:10.1371/journal.pcbi.1008978)
Supplement: S5 File — This PDF file includes the run time of similarity calculation methods and clustering methods. (PDF) [file pcbi.1008978.s005.pdf]

Supplementary File 5

Table 1: Run time of InnerProduct (in seconds) as the number of cells varies.

n_cells	InnerProduct_scaling	InnerProduct_multiplication	InnerProduct_total
100	0.055	0.041	0.095
100	0.046	0.021	0.066
100	0.051	0.044	0.094
100	0.047	0.021	0.067
100	0.054	0.044	0.097
100	0.046	0.021	0.066
100	0.049	0.021	0.069
100	0.042	0.020	0.061
100	0.047	0.021	0.067
100	0.047	0.021	0.067
200	0.102	0.062	0.164
200	0.095	0.069	0.164
200	0.100	0.074	0.174
200	0.093	0.069	0.162
200	0.105	0.093	0.198
200	0.096	0.064	0.160
200	0.095	0.069	0.164
200	0.096	0.070	0.166
200	0.096	0.069	0.165
200	0.121	0.071	0.192
300	0.138	0.099	0.237
300	0.170	0.107	0.277
300	0.148	0.97	0.245
300	0.138	0.094	0.232
300	0.135	0.096	0.231
300	0.138	0.097	0.235
300	0.131	0.093	0.224
300	0.135	0.104	0.239
300	0.132	0.104	0.236
300	0.137	0.097	0.234
400	0.190	0.148	0.338
400	0.193	0.179	0.372

400	0.192	0.149	0.341
400	0.191	0.149	0.340
400	0.193	0.157	0.350
400	0.193	0.150	0.343
400	0.180	0.146	0.326
400	0.193	0.148	0.341
400	0.190	0.151	0.341
400	0.184	0.147	0.331
500	0.251	0.234	0.485
500	0.236	0.227	0.463
500	0.251	0.229	0.480
500	0.248	0.231	0.479
500	0.245	0.227	0.472
500	0.245	0.228	0.473
500	0.251	0.227	0.478
500	0.275	0.234	0.509
500	0.246	0.229	0.475
500	0.245	0.229	0.474
600	0.299	0.306	0.604
600	0.296	0.306	0.601
600	0.289	0.305	0.593
600	0.295	0.304	0.598
600	0.368	0.342	0.709
600	0.285	0.304	0.588
600	0.301	0.307	0.607
600	0.290	0.320	0.609
600	0.286	0.305	0.590
600	0.294	0.305	0.598
700	0.335	0.403	0.738
700	0.434	0.469	0.903
700	0.334	0.431	0.765
700	0.417	0.456	0.873
700	0.335	0.412	0.747
700	0.427	0.468	0.895
700	0.328	0.417	0.745
700	0.328	0.404	0.732
700	0.399	0.494	0.893
700	0.365	0.473	0.838
800	0.384	0.573	0.957
800	0.444	0.596	1.040
800	0.356	0.575	0.931
800	0.360	0.585	0.945
800	0.410	0.557	0.967
800	0.455	0.593	1.048
800	0.395	0.568	0.963
800	0.382	0.571	0.953

800	0.460	0.606	1.066
800	0.355	0.568	0.923
900	0.418	0.841	1.258
900	0.478	0.802	1.279
900	0.477	0.815	1.291
900	0.456	0.796	1.251
900	0.530	0.862	1.391
900	0.569	0.840	1.408
900	0.440	0.742	1.181
900	0.525	0.826	1.350
900	0.563	0.848	1.410
900	0.447	0.823	1.269
1000	0.594	0.966	1.560
1000	0.448	0.930	1.378
1000	0.593	0.999	1.592
1000	0.466	0.932	1.398
1000	0.794	1.084	1.878
1000	0.626	1.050	1.676
1000	0.584	0.993	1.577
1000	0.451	0.948	1.399
1000	0.521	0.926	1.447
1000	0.437	0.961	1.398

Table 2: Run time of HiCRep, fastHiCRep and Selfish (in seconds) as the number of cells varies.

n_cells	HiCRep	fastHiCRep	Selfish
100	265.735	0.070	0.108
100	272.439	0.044	0.108
100	270.759	0.042	0.106
100	262.065	0.042	0.110
100	266.715	0.042	0.107
100	272.348	0.042	0.108
100	253.542	0.042	0.106
100	267.629	0.042	0.106
100	271.510	0.042	0.107
100	276.342	0.042	0.106
200	1063.588	0.108	0.212
200	1080.925	0.104	0.209

200	1071.971	0.104	0.214
200	1180.285	0.117	0.212
200	1088.602	0.107	0.221
200	1143.736	0.113	0.210
200	1116.183	0.094	0.207
200	1096.681	0.103	0.211
200	1094.792	0.095	0.212
200	1121.746	0.114	0.211
300	2476.237	0.188	0.358
300	2426.843	0.186	0.360
300	2616.176	0.186	0.356
300	2480.274	0.187	0.361
300	2412.052	0.177	0.354
300	2516.967	0.183	0.362
300	2513.763	0.189	0.360
300	2415.885	0.188	0.356
300	2431.654	0.196	0.358
300	2417.735	0.191	0.360
400	4551.457	0.281	0.556
400	4426.150	0.284	0.545
400	4486.214	0.284	0.538
400	4469.431	0.324	0.545
400	4481.475	0.327	0.537
400	4406.611	0.275	0.543
400	4479.468	0.278	0.544
400	4379.260	0.287	0.553
400	4521.699	0.278	0.554
400	4317.965	0.277	0.545
500	6951.402	0.406	0.797
500	6920.582	0.390	0.804
500	6944.712	0.418	0.798
500	7001.947	0.414	0.798
500	7024.996	0.437	0.799
500	6992.574	0.416	0.801
500	7102.083	0.417	0.791
500	7111.991	0.389	0.797
500	7107.941	0.405	0.795
500	7092.930	0.417	0.789
600	10360.409	0.560	1.080
600	10194.383	0.532	1.078
600	10335.435	0.520	1.082
600	10624.704	0.587	1.081
600	10358.559	0.548	1.138
600	10550.042	0.529	1.088
600	10463.324	0.526	1.079
600	10363.598	0.555	1.081

600	10421.783	0.532	1.062
600	10275.018	0.543	1.065
700	14391.007	0.688	1.418
700	14193.519	0.689	1.404
700	14317.064	0.704	1.425
700	14340.194	0.694	1.404
700	14439.174	0.735	1.407
700	14372.688	0.748	1.414
700	14296.746	0.695	1.413
700	14488.768	0.685	1.416
700	14220.409	0.698	1.413
700	14600.657	0.694	1.412
800	19086.808	0.864	1.796
800	18932.040	0.949	1.788
800	18785.786	0.840	1.782
800	18861.578	0.848	1.799
800	18990.685	0.885	1.799
800	19033.676	0.881	1.786
800	19113.516	0.882	1.797
800	18722.470	0.864	1.787
800	19110.342	0.882	1.779
800	18803.497	0.856	1.756
900	23930.934	0.942	2.183
900	23912.015	1.029	2.252
900	23866.846	1.011	2.201
900	23636.206	1.020	2.201
900	23921.369	1.064	2.196
900	24004.765	0.974	2.216
900	23963.626	0.977	2.207
900	23891.208	1.047	2.225
900	23942.017	1.015	2.224
900	23713.445	1.056	2.198
1000	31425.658	1.210	2.665
1000	31905.389	1.272	2.683
1000	30513.268	1.448	2.671
1000	28564.591	1.300	2.710
1000	28483.015	1.414	2.811
1000	28376.710	1.297	2.689
1000	26136.064	1.373	2.708
1000	26267.425	1.163	2.572
1000	26033.820	1.351	2.637
1000	25646.701	1.340	2.814

Table 3: Run time of clustering methods (in seconds) for 750 cells.

scHiCluster	InnerProduct_MDS_kmeans	InnerProduct_spectral_clustering
113.427	1.837	1.579
115.074	1.883	1.439
112.784	1.669	1.415
114.773	1.411	1.393
115.903	1.883	1.460
115.041	1.954	1.457
115.279	1.870	1.665
112.820	1.968	1.721
114.017	1.811	1.640
115.094	1.426	1.539
