# Supplementary material for: scHiCTools: A computational toolbox for analyzing single-cell Hi-C data
Source: PLoS Comput Biol. 2021 May 18;17(5):e1008978. doi: 10.1371/journal.pcbi.1008978 (PMC8162587; doi:10.1371/journal.pcbi.1008978)
Supplement: S6 File — This PDF file includes the number of cells and the contacts numbers of Flyamer et al., Collombet et al. and Ramani et al. datasets, and their scatter plots. (PDF) [file pcbi.1008978.s006.pdf]

a

data set	cell type	#cells	median #contacts
Flyamer	oocyte-NSN	40	66075
	oocyte-SN	76	235292
	zygotic-mat	36	110828
	zygotic-pat	35	100428
Collombet	1CSE	99	180771
	2CSE	128	260048.5
	4CSE	146	310121
	8CSE	135	433624
	64CSE	242	132772
Ramani	ML1_MEF	8	7115
	ML1_Patski	329	11469
	ML2_MEF	10	7211
	ML2_Patski	256	12268.5
	ML3_MEF	14	6286
	ML4_Patski	193	9713
	PL2_MEF	125	7164
	PL2_Patski	974	11235.5
	PL1_MEF	57	6966
	PL1_Patski	642	9250.5

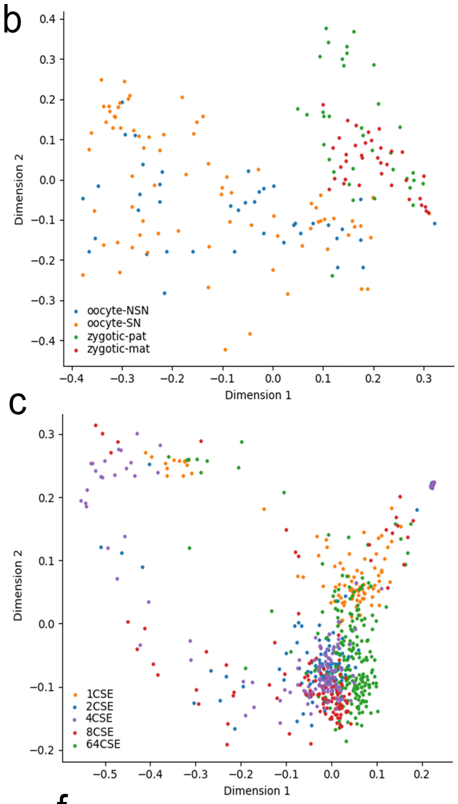

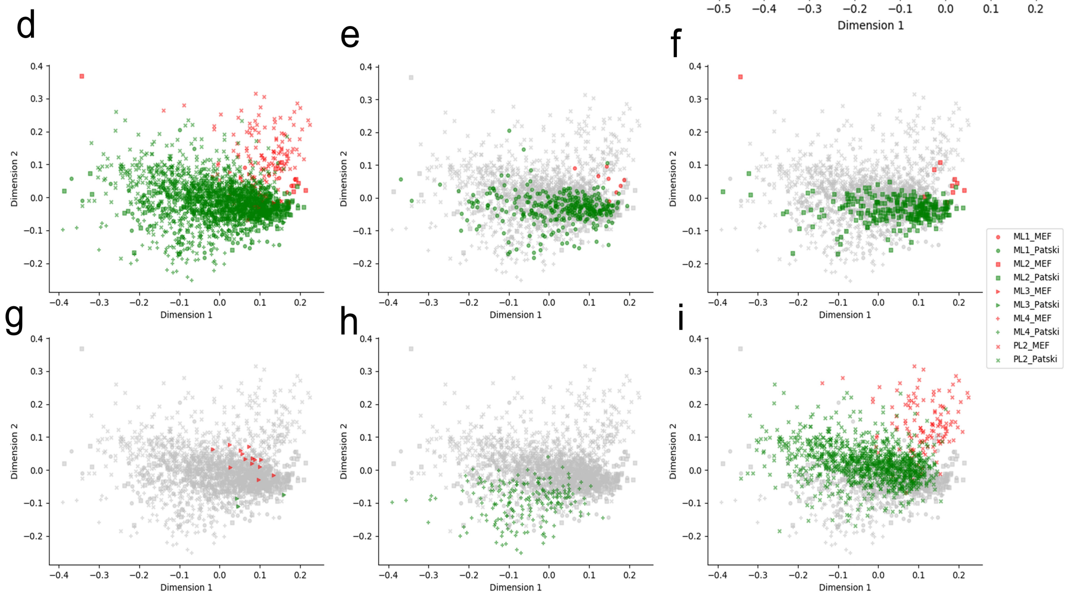

Supplementary File 6: **Applying our toolbox to Flyamer et al. [1], Collombet et al. [19] and Ramani et al. [3] datasets.** (a) The number of cells and their median contact number in each cell of different cell types of single-cell Hi-C datasets other than Nagano et al. Nature 2017 used in our study. (b) Two-dimensional projection from InnerProduct and MDS on Flyamer et al. dataset shows clear separation between oocytes and zygotes. (c) Two-dimensional projection from InnerProduct and MDS on Collombet et al. dataset shows moderate separation of the five cell types. (d) Two-dimensional projection of InnerProduct and MDS on Ramani et al. dataset shows clear separation between MEF and Patski cells. (e-i) Two-dimensional projection of InnerProduct and MDS on Ramani et al. dataset, with the five libraries was colored separately, e:ML1, f:ML2, g:ML3, h:ML4, i: PL2.
